# Unraveling cervical cancer screening dilemmas: histopathological insights from VIA and LEEP at bugando medical centre, Mwanza

**DOI:** 10.1186/s12885-023-11779-1

**Published:** 2024-01-12

**Authors:** Godfrey K. Kaizilege, Edgar Ndaboine, Clotilda Chuma, Fridolin Mujuni, Richard Kiritta, Dismas Matovelo, Oscar Ottoman, Edrick Elias, Nestory Masalu, Benson R. Kidenya, Humphrey D. Mazigo

**Affiliations:** 1 Department of Obstetrics and Gynecology, Weill Bugando School of Medicine, Catholic University of Health and Allied Sciences, Mwanza, P.O. Box 1464, Tanzania; 2 Department of Pathology, Weill Bugando School of Medicine, Catholic University of Health and Allied Sciences, Mwanza, P.O. Box 1464, Tanzania; 3 Department of Oncology, Weill Bugando School of Medicine, Catholic University of Health and Allied Sciences, Mwanza, P.O. Box 1464, Tanzania; 4 Department of Biochemistry and Molecular Biology, Weill Bugando School of Medicine, Catholic University of Health and Allied Sciences, Mwanza, P.O. Box 1464, Tanzania; 5 Department of Parasitology, Weill Bugando School of Medicine, Catholic University of Health and Allied Sciences, Mwanza, P.O. Box 1464, Tanzania

**Keywords:** VIA, LEEP, Precancerous lesions, Cervical cancer

## Abstract

**Background:**

The single-visit strategy, also known as the “screen-and-treat” approach, is widely used to screen for cervical cancer in low- and middle-income countries. The screen-and-treat approach leads to unnecessary or inadequate treatment. Thus, a study was conducted to determine the histopathological patterns of aceto-white lesions on visual inspection with acetic acid (VIA) in patients who underwent a Loop Electrosurgical Excision Procedure (LEEP) at Bugando Medical Centre between January 2016 and December 2020.

**Method:**

A 5-year retrospective cross-sectional case record review was conducted on 329 women who had LEEP at Bugando Medical Centre following a positive VIA cervical screening test. A standard data abstraction form was used to collect patient information. Missing client information records and LEEP without histopathological results were exclusion criteria. For statistical analysis, STATA version 15 was used; in descriptive statistics, frequency, mean, and standard deviation were used. The Chi^2^ and Fisher’s exact tests were used to investigate the relationship between patient characteristics and histopathological patterns, and a *P*-value of 0.05 was considered statistically significant in multinomial models.

**Results:**

This study looked at 329 patients who had LEEP following a VIA positive but were not eligible for cryotherapy. Our study participants had a mean age of 40 ± 8.2 SD. There were 203 (61.7%) patients with benign lesions, including 4 with schistosomiasis and 2 with cervical tuberculosis. The precancerous lesions were discovered in 100 cases (30.4%), and 26 (7.9%) already had invasive cervical cancer. Out of 100 patients with precancerous lesions, 58 (17.6%) and 42 (12.8%) have high- and low-grade squamous intraepithelial (HSIL and LSIL) lesions, respectively. The presence of a precancerous lesion was found to be associated with age 31–40 years (*P*-value 0.042) and HIV positivity (*P*-value 0.004).

**Conclusion:**

Most patients in this study had benign cervical lesions, which do not require LEEP treatment. Nonetheless, a considerable percentage of invasive cervical malignancies and rare benign diseases such as schistosomiasis and cervical tuberculosis were identified. A screen-and-treat approach within well-equipped tertiary hospitals like Bugando Medical Centre should explore alternative options instead of relying solely on straight LEEP.

## Introduction

There are an estimated 604,000 newly diagnosed cases of cervical cancer and 342,000 deaths associated with the disease worldwide, which ranks fourth for both incidence and mortality. Approximately 85–90% of patients are from low- and middle-income countries, including Sub-Saharan Africa, South-East Asia, and Latin America, among people from socioeconomically weaker sections of society [1, 2]. Cervical cancer is the leading cause of female cancer in Tanzania, with a 59.1 per 100,000 incidence rate and a mortality rate of 42.1 per 100,000 [3]. Cervical cancer screening has remained an important strategy for cervical cancer prevention by detecting and treating precancerous lesions as early as possible. The WHO-approved cervical cancer screening methods include cytology (conventional or liquid-based), HPV DNA testing, and visual inspection with acetic acid (VIA) [4]. Tanzania adopted the WHO-recommended screen-and-treat approach to minimize cervical cancer. In this strategy, a precancerous lesion is treated based on a screening test called visual inspection with acetic acid (VIA), and therapy is given on the same day [4, 5].

VIA screening has been widely implemented in opportunistic settings in many low-income countries in Sub-Saharan Africa. A screen-and-treat approach for screening with rapid diagnosis and treatment improves coverage, eliminates follow-up visits, and makes screening more time and cost-efficient in low-resource settings [6]. Treatment options available for cervical precancerous lesions include cryotherapy, loop electrosurgical excision procedure (LEEP), and cold knife conization (CKC) [7]. Several studies done assessing the histopathological patterns of VIA-positive cervical lesions after the application of acetic acid followed by a biopsy or LEEP treatment found that the majority of the specimens taken had normal cervical tissue, chronic cervicitis, and low-grade squamous intraepithelial lesions (LSIL) of about 65–75% and did not require treatment [7–9].

Visual inspection with acetic acid compared with other screening methods like cytology and HVP-DNA testing has been associated with high sensitivity ranging between 74 and 100% but with low specificity ranging between 48 and 53% [9, 10]. This contributes significantly to overtreatment, studies done at Nairobi University in Kenya and Kitwe Teaching Hospital in Zambia showed an overtreatment rate of 65% and 75% respectively [10, 11].

Controversies exist regarding the management of cervical intraepithelial neoplasia in a screen-and-treat approach when utilizing VIA as the sole cervical screening method. Some studies show screening results that are comparable to other methods like cytology and HPV-DNA testing, while others report differing results. Consequently, some recommendations advocate for combining VIA with other methods to enhance screening accuracy [10, 12]. Results of another method LEEP has been used as a treatment option for VIA-positive patients in the screen-and-treat approach, either in a single-visit approach or a multiple-visit approach, in most facilities in Tanzania. In the “screen-and-treat approach,” the decision to treat is based only on a positive primary screening test. While in the “screen, triage, and treat approach,” the decision to treat is based on a positive primary screening test followed by a positive second test (a “triage” test); the latter is not widely utilized [12–14].

Despite its usefulness, LEEP has been associated with several complications grouped into early and long-term/late complications. Early complications include heavy bleeding during the procedure that necessitates suturing or /and transfusion, infection, and damage to the vaginal walls. Late complications include cervical stenosis, and cervical incompetency that may lead to poor pregnancy outcomes like abortion and preterm delivery [15]. Therefore, the decision to use LEEP for treating precancerous lesions should be made under solid evidence of histology especially in young patients. As a consequence of this, a study was carried out to determine the histopathological patterns of aceto-white lesions on visual inspection with acetic acid in patients who had undergone a LEEP in a screen-and-treat approach at Bugando Medical Centre between January 2016 and December 2020.

## Materials and methods

A retrospective cross-sectional study was done at Bugando Medical Centre (BMC) in Mwanza, Tanzania, in the Department of Oncology, cancer screening unit, from January 2016 to December 2020. Medical records of patients who underwent LEEP treatment in the screen-and-treat approach were retrieved from the MTUHA (Tanzanian manual method of data storage that uses a tally sheet at the facility level) database using a data extraction sheet. Histology results were obtained from the histopathology department in the electronic database and matched with patients using the unique screening identification number.

Since 2016, the oncology cancer screening section has provided cervical cancer screening using a “screen-and-treat” approach in which clients are screened with acetic acid and treated concurrently if the test is positive. For VIA-positive-screened clients, cryotherapy or LEEP is commonly offered. All LEEP procedures were performed by trained medical doctors, typically registrars or gynecologists from the Obstetrics and Gynecology department, except cryotherapy, which is commonly administered by trained nurses or doctors. A large acetowhite lesion occupying more than 75% of the cervix, an acetowhite lesion that extends to the endocervix, recurrent VIA positivity after one year of cryotherapy, and lesions that could not be covered with a cryoprobe were the criteria for LEEP. Collected LEEP specimens or tissue biopsies are histologically evaluated at the central pathology laboratory after it is obtained. As per protocol and standard operating procedures (SOP), LEEP biopsies are usually kept in 10% neutral buffered formalin for a while before being processed and embedded in paraffin wax. Finally, 5-micron-thick slices of tissue are sectioned by rotary microtome and stained with Harris-Hematoxylin and Eosin stains. Then, two registered pathologists looked at least five slides of each specimen using an Olympus CX21 microscope to make the final pathological anatomical diagnosis of the lesion. The final pathology report and diagnosis were given back to the patient in the screening room so that they could give feedback and, if necessary, decide on further treatment. All tissue sections with diseases of interest are photographed using a camera connected directly to the microscope.

Our study’s inclusion criteria encompassed all patient records who underwent VIA screening with 5% acetic acid, received LEEP treatment during screen-and-treat at BMC, had well-documented biopsy reports in the electronic database, and had comprehensive clinical information recorded in the MTUHA registry. Our exclusion criteria comprised patients who had undergone cervical cancer biopsies, those with incomplete information in the LEEP biopsy report, and patients already diagnosed with cervical cancer. The study criteria were met by 329 study participants during the study period. The cancer screening register (MTUHA) was used to extract patient demographics such as residence, age, parity, HIV status, number of screenings, type of screening, and treatment received.

All data from this study were entered into Microsoft Excel 2013 before being uploaded to STATA version 15 for analysis. To summarize the categorical data, proportions, and percentages were used, while the mean and standard deviation were used to summarize the continuous data. Chi-square and Fisher’s exact tests of association were used to determine whether or not patient characteristics and histopathological findings were correlated. In multinomial models, statistical significance was assumed for any variable with a *P*-value less than 0.05.

## Results

### Patient’s characteristics

A total of 329 participants met the inclusion criteria and were recruited for the study; the participants’ mean age was 40 ± 8.24 SD. Almost half of our participants, 154 (46.7%), were HIV positive, and 140 (42.6%) were referred for LEEP treatment from the lower facilities following a positive VIA who were disqualified for cryotherapy. Other patient demographics are indicated in Table 1 below.

**Table 1:** Characteristics of the patients who had a positive visual inspection with acetic acid (VIA) screening test underwent loop electrosurgical excision procedure (LEEP)

Patient characteristics	Number (n = 329)	Percentage (%)
***Age***		
21–30 years	44	13.4
31–50 years	256	77.8
≥51 years	29	8.8
***Parity***		
Nulliparous	14	4.3
Para 1–4	203	61.7
Para ≥ 5	112	34.0
***Residence***		
Urban	212	64.4
Rural	117	35.6
***HIV status***		
HIV positive	154	46.8
HIV negative	175	53.2
***LEEP treatment provider***		
Medical officer	239	72.6
Gynecologist	90	27.4
***Type of visit***		
First visit for VIA	125	37.9
First follow-up a year after treatment with CRYO/LEEP	26	7.9
A follow-up visit after VIA negative	38	11.6
Client with VIA positive referred for CRYO/LEEP	140	42.6

### Histopathology pattern of positive visual inspection with acetic acid (VIA) screening test and underwent loop electrosurgical excision procedure (LEEP)

Among 329 participants, 100 (30.4%) had squamous intraepithelial lesions (high- and low-grade squamous intraepithelial lesions). Also, 26 (7.9%) participants had cancerous lesions. A total of 203 (61.7%) participants had benign lesions. However, 10 (3.1%) of our participants who underwent LEEP had a normal cervix as narrated in Table 2.

**Table 2:** Histopathology pattern of positive visual inspection with acetic acid (VIA) screening test and underwent loop electrosurgical excision procedure (LEEP)

*Lesion type*	*Histopathological results*	*frequency (n)*	*Percentage (%)*
**Benign lesion**		**203**	**61.7**
	Chronic cervicitis	155	47.1
	Squamous metaplasia without atypia	12	3.7
	Normal cervical	10	3.0
	Nabothian cyst	7	2.1
	Cervical polyp	6	1.8
	Cervical leiomyomatous	5	1.5
	Cervical schistosomiasis	4	1.2
	Condyloma acuminate	2	0.6
	Tuberculosis	2	0.6
**Precancerous**		**100**	**30.4**
	Low-grade squamous intraepithelial lesion	42	12.8
	High-grade squamous intraepithelial lesion	58	17.6
**Cancerous lesion**		**26**	**7.9**
	Micro-invasion	13	4.0
	Invasive squamous cell carcinoma	9	2.7
	Adenosquamous carcinoma	2	0.6
	Adenocarcinoma	2	0.6

### The correlation between LEEP biopsy histopathological patterns and patient characteristics

HIV status and patient age (31–40 years) had a significant influence on the LEEP outcome, according to chi-square analysis and multinomial logistic regression. With a *P*-value of 0.004, HIV-positive patients were more likely to have a cervical precancerous lesion high-grade than HIV-negative patients. The age of the patient was another factor that showed a significant association with the presence of a precancerous lesion. With a *P*-value of 0.042, patients aged 31–40 years have significantly more precancerous lesions than other age groups as indicated in Table 3.

**Table 3:** The correlation between LEEP biopsy histopathological patterns and patient characteristics

*Patient characteristics*	*Cervical cancer*	*Pre-cancerous*	*Benign lesion*	*Total*	*Chi2*	*P*-value	*Multinomial logistic regression*	
	n (%)	n (%)	n (%)				***R (95%CI)***	***P-value***
***HIV status***								
HIV positive	14 (53.9)	61 (61.0)	79 (38.9)	154	13.684	0.001	**2.1[1.28–3.51]**	**0.004**
HIV negative	12 (46.2)	39 (39.0)	124 (61.1)	175				
***Parity***								
Nulliparous	2 (7.7)	6 (6.0)	6 (2.9)	14	-*	0.311	–	–
Para1-4	13 (50.0	64 (64.0)	126 (62.1)	203				
Para ≥ 5	11 (42.3	30 (30.0)	71 (34.9)	112				
***Age***								
21–30	3 (11.5)	13 (13.0)	28 (13.8)	44			2.2 [0.62–7.83]	0.220
31–40	9 (34.6)	52 (52.0)	66 (32.5)	127			**3.3[1.04–10.37]**	**0.042**
41–50	11 (42.3)	31 (31.0)	87 (42.9)	129	-*	0.035	1.7 [0.52–5.31]	0.383
≥51	3 (11.5)	4 (4.0)	22 (10.8)	29			–	–
***Type of visit***								
First visit for VIA	7 (5.6)	30 (24.0)	88 (70.4)	125				
First follow-up a year after treatment with CRYO/LEEP	2 (7.7)	12 (46.2)	12 (46.2)	26				
A follow-up visit after VIA negative	3 (7.9)	11 (29.0)	24 (63.2)	38	-*	0.167	–	–
Client with VIA positive referred for CRYO/LEEP	14 (10.)	47 (33.6)	79 (56.4)	140				
***LEEP treatment provider***								
Medical officer	21 (80.8)	65 (65.0)	153 (75.4)	239	–*	0.102	–	–
Gynecologist	10 (8.9)	37 (32.7)	66 (58.4)					

-* *P*-value was calculated by Fisher’s exact test

## Discussion

To the best of our knowledge, this is the first study of LEEP treatment biopsies reviewed at Bugando Medical Centre and in Tanzania in general. In Tanzania, cervical cancer screening adheres to the 2013 WHO guidelines, which recommend a screening strategy involving VIA followed by immediate treatment with cryotherapy or LEEP during the same session in resource-limited settings where HPV testing is not feasible [4, 5]. In this study, 7.9% of women treated with LEEP had cervical cancer; this is the same as a study conducted between 2015 and 2018 in Zambia’s tertiary hospital, where the prevalence was 8% [11]. Other studies have shown different prevalence values for cervical cancer, ranging from 1.9–6% [16–18]; the differences observed may be affected by study design, study population, and HIV seropositivity. This demonstrated the importance of reviewing LEEP biopsies and allowed treatment to begin at the earliest stage of the disease. Additionally, all 26 women diagnosed with early cervical cancer were treated immediately with radical hysterectomy upon receiving their LEEP results.

According to LEEP biopsy, 30% of study participants had low- or high-grade cervical intraepithelial lesions (LSIL or HSIL), with a significant number having high grades. LEEP was sufficient as a method for preventing the progression of cervical cancer. However, 46.8% of participants were HIV-positive, placing them at the highest risk for the progression of the disease to invasive cancer. For many years, HIV-infected women’s risk for cervical cancer remained high and stable, and incidence did not decrease with improving CD4 cell counts. Methods to improve cervical cancer screening sensitivity and specificity have demonstrated promising results, particularly when combined with HR-HPV DNA testing. In immunocompromised women, primary HPV DNA screening proves to be more specific and effective. Recent HPV vaccines have therapeutic potential and may help prevent cervical cancer in individuals with multiple and persistent HPV infections [12]. In a recent multicenter study conducted in Italy, involving the review of 2,966 patient charts following conization, 5.5% of patients exhibited HPV persistence. Within this group, 10.4% experienced a recurrence of CIN2 + during the 5-year follow-up. An elevated risk of recurrence was linked to HR-HPV infection between 6 months and one-year post-procedure, along with the presence of positive surgical margins for CIN2 +  [19, 20]. This indicates that persistent HPV infection significantly increases the risk of recurrence. This is supported by the fact that nearly 62% of our patients were on follow-up visits, having previously tested negative with VIA or having been referred with persistent VIA positivity after treatment. If left untreated, persistent HPV infection can ultimately progress to invasive cancer. Therefore, the well-established and effective approach to preventing cervical cancer involves screening and treating preinvasive stages.

Almost 65% of study participants who received LEEP treatment had benign cervical lesions and a normal cervix, with the majority suffering from chronic cervicitis (47%). This group did not need LEEP treatment in the first place, given the complications that may arise from LEEP and the costs of unnecessary treatment; however, the study did not establish the cause of chronic cervicitis. Most studies show that Neisseria gonorrhoeae, Chlamydia trachomatis, Herpes simplex virus (HSV), Human papillomavirus (HPV), and Trichomonas vaginalis, as well as other unknown factors, are the main causes of chronic cervicitis [21–23]. More research is needed in this area to determine the primary local cause of chronic cervicitis so that effective treatment can be provided. Additionally, the LEEP biopsy detected two granulomatous infection conditions, schistosomiasis, and tuberculosis; however, despite the rarity of this condition on the uterine cervix, being in an endemic area makes it possible for the disease to spread to the cervix. Several studies have found that these infections can also be found in the cervix of the uterus [24, 25]. Female genital schistosomiasis is known to be endemic in the study area and community studies have reported a prevalence of 5% [26]. All six patients with these infections received appropriate treatment and, upon review, were declared cured.

The correlation between LEEP biopsies, histopathological patterns, and patient characteristics demonstrates that HIV seropositivity and age of the study participant have a significant impact on the development of a cervical precancerous lesion (*P*– 0.004 and 0.042 respectively). This finding is consistent with other studies because low HPV immunity and the natural history of cervical cancer both follow the same path [27, 28].

The review of LEEP biopsies performed at our Center as part of the “screen-and-treat approach” with LEEP if cryotherapy is not an option revealed a flaw that resulted in overtreatment in 65% of study participants with benign or normal cervical conditions. It is now time to implement a new WHO recommendation of 2021: the “screen, triage, and treat approach,” in which the decision to treat is based on a positive primary screening test, followed by a positive second test (a “triage” test), with a histologically confirmed diagnosis [12]. Tanzania and other low- and middle-income countries can consider implementing the new WHO strategy, particularly within well-equipped teaching university hospital facilities equipped with screening rooms, a pathology lab, and a sufficient number of trained staff. The previous screen-and-treat approach should primarily be retained for low-capacity primary care facilities.

### Study strength

This was the first study at Tanzania’s Bugando Medical Centre to examine the treatment of precancerous lesions with LEEP after a positive VIA and ineligibility for cryotherapy in a setting where a single-visit approach (a “screen and treat” approach) is common. The study highlighted the significance of precancerous and cancerous lesion detection as well as the extent of overtreatment.

### Study limitation

Because this was a single-center tertiary hospital study, the magnitude of overtreatment may have been overestimated. This was a retrospective study that extracted information from the registry that could have been influenced by patient recording.

## Conclusion

The majority of patients in this study had cervical lesions that were benign or normal, which means that they did not require LEEP. Despite this, a sizeable proportion of invasive cervical cancers as well as uncommon benign diseases such as schistosomiasis and cervical tuberculosis were discovered. The correlation between LEEP biopsies, histopathological patterns, and patient characteristics demonstrates that HIV seropositivity and age of the study participant have a significant impact on the development of a cervical precancerous lesion (P– 0.004 and 0.042 respectively).

## Recommendation

There is a need to make some adjustments to the screening process for cervical cancer so that we can cut down on unnecessary treatment. Tertiary hospitals such as Bugando Medical Centre should consider a colposcopic guided biopsy as an alternative to straight LEEP for patients with an acetowhite lesion on the VIA test, as the new WHO guideline recommends.

## Data Availability

The dataset used and/or analyzed during the current study is available from the corresponding author upon request.
